# Drinking Green Tea: Despite the Risks Due to Mycotoxins, Is It Possible to Increase the Associated Health Benefits?

**DOI:** 10.3390/toxins13020119

**Published:** 2021-02-05

**Authors:** Ricardo Assunção, Magdalena Twarużek, Robert Kosicki, Carla Viegas, Susana Viegas

**Affiliations:** 1 CESAM—Centre for Environmental and Marine Studies, University of Aveiro, 3810-193 Aveiro, Portugal; 2 Food and Nutrition Department, National Institute of Health Dr. Ricardo Jorge, 1649-016 Lisbon, Portugal; 3 NOVA National School of Public Health, Public Health Research Centre, Universidade NOVA de Lisboa, 1600-560 Lisbon, Portugal; carla.viegas@estesl.ipl.pt (C.V.); susana.viegas@ensp.unl.pt (S.V.); 4 Department of Physiology and Toxicology, Faculty of Biological Sciences, Kazimierz Wielki University, Chodkiewicza 30, 85-064 Bydgoszcz, Poland; twarmag@ukw.edu.pl (M.T.); robkos@ukw.edu.pl (R.K.); 5 H&TRC-Health & Technology Research Center, ESTeSL-Escola Superior de Tecnologia da Saúde, Instituto Politécnico de Lisboa, 1990-096 Lisbon, Portugal; 6 Comprehensive Health Research Center (CHRC), 1169-056 Lisbon, Portugal

**Keywords:** green tea, mycotoxins, polyphenols, EGCG, risk–benefit assessment

## Abstract

Tea has been consumed for thousands of years. Despite the different varieties, particular emphasis has been placed on green tea (GT), considering the associated health benefits following its regular consumption, some of which are due to its polyphenol constituents, such as epigallocatechin-3-gallate (EGCG). Tea is not prone to the growth of microorganisms, except fungus, when proper storage, handling, and packing conditions are compromised. Consequently, mycotoxins, secondary metabolites of fungi, could contaminate tea samples, affecting human health. In the present study, we aimed to assess the balance between risks (due to mycotoxins and high levels of EGCG) and benefits (due to moderate intake of EGCG) associated with the consumption of GT. For this, 20 GT samples (10 in bulk and 10 in bags) available in different markets in Lisbon were analyzed through a LC–MS/MS method, evaluating 38 different mycotoxins. Six samples revealed detectable values of the considered toxins. Current levels of mycotoxins and EGCG intake were not associated with health concerns. Scenarios considering an increasing consumption of GT in Portugal showed that drinking up to seven cups of GT per day should maximize the associated health benefits. The present study contributes to the future establishment of GT consumption recommendations in Portugal.

## 1. Introduction

Tea has been consumed for thousands of years. In Portugal, despite modest production, tea consumption is also increasing, mostly due to influences from other cultures [1]. The largest tea producers in the world are China, India, Sri Lanka and Turkey, with China and India covering about 43% and 22% of the world’s production, respectively [2]. In Portugal, two main producers can be identified. These are located on the island of São Miguel, in Azores—one with larger scale production, named Gorreana, and another with a reduced production volume, called Porto Formoso [1].

The most commonly consumed types of tea worldwide are black, white, oolong, green, and Puerh (postfermented) tea [3]. Due to healthy lifestyle trends associated with several developed studies that have indicated a wide variety of health benefits, green tea consumption has increased [4,5]. From the already claimed health benefits, several reports have already identified: the reduction in the occurrence of cardiovascular diseases [6], the inhibition of matrix metalloproteinases [7], the use as stimulant, the regulation of body temperature and antimicrobial activity [8], regulation of blood sugar, and promotion of digestion [9]. These health benefits are usually associated with green tea components such as vitamins, microelements, essential oils, and polyphenols [10]. From all these beneficial food components, polyphenols have received particular interest, being the most relevant the catechins and the flavonols. Previous authors have demonstrated that these polyphenols have the capacity to alter the pathogenesis of some chronic diseases due to their antioxidant, anti-inflammatory, antiproliferative, antimutagenic, antibacterial, and antiviral characteristics providing protection against cardiovascular disease, hyperglycemia, metabolic disorders, and some cancers [11,12].

Catechins can be found in high concentrations in fresh tea leaves, rock-rose leaves, broad beans, red wine, black grapes, strawberries, and apricots [13]. However, the most important dietary source of catechins is green tea [14]. Regarding the antioxidant efficacy of catechins, it is important to mention that this efficacy is due to direct mechanisms—scavenging reactive oxygen species (ROS), chelating metal ions—and indirect mechanisms—inducing antioxidant enzymes, inhibiting pro-oxidant enzymes, and producing phase II detoxification enzymes (e.g., glutathione) and antioxidant enzymes (e.g., glutathione peroxidase) [14].

Catechins also have important roles in preventing oxidative stress-caused diseases such as cancer, neurodegenerative diseases—Parkinson’s disease and Alzheimer’s disease —cardiovascular diseases, and diabetes, since all these diseases are linked to changes in oxidant–antioxidant balances and free radical damage [15,16]. Therefore, due to their antioxidant properties, catechins may be particularly valuable in preventing and protecting from pathologies associated with oxidative stress [16,17]. However, green tea extracts, particularly (-)-epigallocatechin-3-gallate (EGCG), the most relevant catechin in green tea, have been associated with cases of hepatotoxicity. In 2018, a European Food Safety Authority (EFSA) panel indicated that there is scientific evidence from interventional clinical trials that the intake doses of EGCG equal or above 800 mg/day taken as a food supplement induce a statistically significant increase in serum transaminases in treated subjects when compared with a control group [18].

Concerning microbiological contamination, it is expected that tea contains a reduced level of microorganisms due to its low water activity and, consequently, the reduced risk related to the growth of microorganisms [19]. However, inappropriate storage, handling, and packing conditions of tea can increase the possibility of fungal contaminations [20,21] and/or growth [3]. Indeed, microbial contamination was already observed in samples of bulk and bags of green tea marketed in Lisbon [22]. This study reported a significant reduction in bacterial contamination after boiling; however, fungal presence with toxigenic potential was reported before and after boiling [22].

Additionally, mycotoxins can also be present in tea due to the fungal contamination. Mycotoxins are natural toxins produced by specific fungi that can grow on a variety of harvests [23]. Mycotoxin production in tea can occur at any production stage such as tea bush cultivation, harvest, processing, and storage. Poor agricultural procedures, improper processing, drying, packaging, storage, and transport conditions stimulate fungal growth, increasing the risk of mycotoxin contamination [3,20].

A subtropical climate, being favorable for tea farming, is also adequate for toxigenic mold growth. Aflatoxins and ochratoxin A are two the most toxic mycotoxins already detected in tea samples, but fumonisins, deoxynivalenol, and enniatins have also been reported in previous studies [20,24].

Even when considering the beverage only, it is important to consider that several factors can influence the mycotoxin transfer from the raw tea such as the raw tea contamination level, mycotoxin thermal stability, and its ability to transfer from the matrix into aqueous infusions. Definitely, brewing is incapable of destroying common mycotoxins in a substantial manner [25,26].

Nevertheless, mycotoxins in tea are not properly regulated, except in some countries—namely, in Customs Union countries (Armenia, Belarus, Kazakhstan, Kyrgyzstan, and Russia)—for aflatoxin B1 in raw tea (5 µg/kg); in Argentina, established limits for aflatoxin B1 (5 µg/kg) and total aflatoxins in materials used for herbal tea infusions are 20 µg/kg [27]. Upper limits for a category such as “all foods” have been fixed in several Asian countries [3]. In the EU, a regulation setting the maximum levels for mycotoxins in foodstuffs does not specifically consider tea [28].

Over recent years, combined assessments of risks and benefits associated with different food components, such as, e.g., hazardous agents, nutrients, as well as single foods and whole diets, have been carried out, resulting in the establishment of “risk-benefit assessment” (RBA) as a new multidisciplinary and integrated scientific discipline [29,30,31,32]. The balance between risks and benefits established by the RBA is very relevant to the food-related authorities, contributing to the development of food policies and consumer guidance and recommendations, to businesses developing new food products, and to consumers considering dietary changes [33].

Taking into account the potential health benefits associated with the consumption of green tea [4,18], mainly due to the intake of catechins, and the potential health risks due to mycotoxin contamination and/or high levels of intake of catechins, an adequate balance regarding these two aspects is needed. The present study intended to evaluate the occurrence levels of mycotoxins in green tea samples marketed in Portugal, to assess the balance between risks (due to mycotoxins and high levels of catechins) and benefits (due to moderate intake of catechins) associated with the consumption of green tea, and to assist future research regarding the risk–benefit assessment of green tea, contributing to support consumption recommendations of green tea in Portugal.

## 2. Results

### 2.1. Mycotoxins Occurrence in Tea Samples

**Figure 1:**
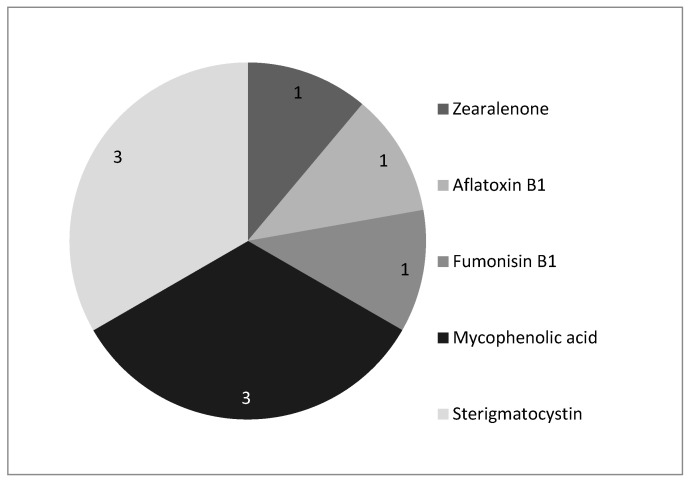
Number of bulk samples positive (above limit of detection (LOD)) for mycotoxins.

Twenty samples (10 bulk and 10 bag samples) were analyzed for the presence of mycotoxins. In the bulk samples, five samples (50%) showed mycotoxin contamination (presenting at least one mycotoxin) and, in one of the samples, five mycotoxins were simultaneously detected—namely, zearalenone (one sample, 9.0 ng/g), aflatoxin B1 (one sample, <2.4 ng/g), fumonisin B1 (one sample, 15.8 ng/g), mycophenolic acid (three samples, <16.2, 154.5 and 170.4 ng/g), and sterigmatocystin (three samples, all <2.4 ng/g) (Figure 1).

In bag samples, only one mycotoxin (mycophenolic acid) was detected in six samples (60%). The values ranged between <16.2 (limit of detection (LOD)) and 66.8 ng/g.

### 2.2. Mycotoxins and Catechins Estimated Intake

Table 1 summarizes the estimated intake of mycotoxins (aflatoxin B1 (AFB1), fumonisin B1 (FB1), zearalenone (ZEA), and sterigmatocystin (STER)) and catechins (EGCG) and the associated risk. Taking into consideration the reported consumption of green tea in Portugal (corresponding to the current situation), or the alternative scenarios considered in the present study (corresponding to the hypothetical situations), the probable intake of mycotoxins and EGCG was calculated, as well as the risk associated with calculated level of exposure.

Considering the current consumption of green tea in Europe (as described by the EFSA, 2018), the estimated levels of intake of mycotoxins ranged between 0.00002 (AFB1 and STER) and 0.0019 ng/kg bw/day (FB1). For the hypothetical scenarios, the highest estimated intake was determined for FB1. Considering the associated risk, none of the current and hypothetical scenarios revealed a level of intake that represents a concern for public health—i.e., Margins of exposure (MOEs) of AFB1 and STER intakes were all significantly above 10,000 (highlighted in green in the Table 1); Hazard quotients (HQs) of FB1 and ZEA intakes were all significantly below 1 (highlighted in green in Table 1).

Regarding catechins, EGCG estimated intake considering the current EU consumption ranged between 86.0 and 321.2 mg/day. EGCG intakes in current consumption (for the minimum and maximum consumption levels) were below the reference level of no hepatotoxicity (800 mg/day), suggesting that these consumption levels are associated with the health benefits usually associated with the intake of catechins. However, for the hypothetical scenarios higher than seven cups per day, it is expected that the intake levels of EGCG would exceed the referred no hepatotoxicity level, and consequently, should be avoided (highlighted in red in the Table 1). According to these results, seven cups of green tea correspond to the highest quantity that could be drunk without expecting health consequences.

## 3. Discussion

Generally, tea consists of polyphenols, caffeine, minerals, and trace levels of vitamins, amino acids, and carbohydrates [34]. From all the varieties of tea, green tea has gained particular relevance due to the significant health benefits assigned to its reach content in polyphenols (i.e., catechins and flavonols)—namely, through their antioxidant, anti-inflammation, anticancer, anticardiovascular, antimicrobial, antihyperglycemic, and antiobesity properties [35]. The health benefits of green tea, in particular EGCG, were widely investigated. In addition to green tea, EGCG can be found in chocolate (600 mg/L), red wine (300 mg/L), and fruits—e.g., apricots or cherries (250 mg/kg fresh weight) [36]. Catechins are also widespread in vegetables such as broad beans and plant-derived products such as wine [37]. Among all catechins that can be found in food, catechin (C), epicatechin (EC), epigallocatechin (EGC), epicatechin gallate (ECG), and EGCG are the most prevalent. However, after brewing green tea, catechins could undergo conversion to suitable epimers, such as epigallocatechin (ECG) to gallocatechin (GC) and EGCG to gallocatechin gallate (GCG) [38].

Harmful effects of green tea overconsumption were also reported and are mainly due to three main reasons: its caffeine content, the presence of aluminum, and the effects of tea polyphenols on iron bioavailability [39]. Indeed, some studies revealed the ability of tea plants to accumulate high levels of aluminum [40]. Furthermore, green tea catechins may present an affinity for iron, and infusions can cause an important decrease in the iron bioavailability from the diet [41]. In 2018, Hu et al. performed a systematic review of published toxicology and human intervention studies aiming to characterize potential hazards associated with consumption of green tea and its preparations. In this study, a safe intake level of 338 mg EGCG/day was recognized for adults which was derived from toxicological and human safety data for tea preparations ingested as a solid bolus dose [42].

Recently, the EFSA has stated that, from the clinical studies reviewed, there is no evidence of hepatotoxicity below 800 mg EGCG/day up to 12 months [18]. The EFSA panel also concluded that catechins from green tea infusion, prepared in a traditional way, and reconstituted drinks with an equivalent composition to traditional green tea infusions are generally considered to be safe according to the presumption of safety approach considering the intake corresponding to the reported consumption in European Member States [18]. However, the health effects linked with this EGCG dosage (<800 mg EGCG/day up to 12 months) still need to be demonstrated.

Although there are several publications in agreement with the long history of safe consumption of large quantities of green tea as a beverage by humans without any reported negative health effects [42,43], with the current knowledge, some considerations concerning consumption rates should be highlighted.

In the present study, the balance between risks (due to mycotoxins and high levels of EGCG) and benefits (due to moderate intake of EGCG) associated with the consumption of green tea was assessed in an attempt to shed light on the suitable consumption rates of green tea. In fact, in addition to the health effects induced by polyphenols usually present in green tea, other aspects should be considered in terms of the impact drinking this beverage on consumers’ health. As evidenced in the present study, mycotoxins could contaminate green tea, constituting a human exposure source to mycotoxins [11]. Likewise, using a multimycotoxin LC–MS/MS method, Pallarés et al. (2017) analyzed 16 mycotoxins in 44 tea samples, including 10 samples of green tea [44]. Contrary to the results obtained in the present study, which shows the presence of five different mycotoxins, Pallarés et al. (2017) revealed that enniatin B was the only mycotoxin detected in the green tea samples analyzed (2 out of 10 samples) at levels below the quantification limits (limit of quantification (LOQ) = 0.2 µg/L) [44]. Aflatoxins, deoxynivalenol, nivalenol, HT-2 and T-2 toxins, zearalenone, ochratoxin A, and beauvericin were not detected in any sample. Considering food supplements of green tea, Martínez-Domínguez et al. showed that the analyzed samples were contaminated by aflatoxin B1 (one positive sample, 5.4 µg/kg) [45]. Aflatoxins B2, G1, G2, deoxynivalenol, fumonisin B1 and B2, HT-2 and T-2 toxins, ochratoxin A, and zearalenone were not detected in the considered samples [45]. It is important to mention in this context that the use of the LC–MS/MS technique (characterized by high sensitivity) in the presented study allowed for a significant simplification of the procedure of sample preparation. An additional advantage is the possibility of simultaneous determination of many mycotoxins belonging to different groups of compounds with different structures. Many mycotoxins determined with the use of conventional detectors, e.g., fluorescence, require chemical derivatization (e.g., aflatoxins, fumonisins). In tandem mass spectrometry, this step does not occur. Another advantage of using LC–MS/MS is obtaining additional identification points for a given compound, based not only on the chromatographic retention time, but also on the mass of precursor and product ions. The influence of matrix components on the measurement signal is the main disadvantage of using the LC–MS/MS technique in food analysis. Additional disadvantages are the associated high cost of purchasing the equipment and the need to have qualified personnel to operate it.

The results obtained in the present study revealed that the risk due to the current exposure to mycotoxins through green tea consumption in Portugal is not associated with health concern (MOE > 10,000 and HQ < 1 in the current estimated exposure). Nevertheless, interesting results were described by some authors regarding the potential protective effects of EGCG on the toxicity associated with mycotoxins. Marnewick et al. showed that green tea presented chemoprotective properties against cancer promotion induced by fumonisin B1 in rat liver [46]. Sugiyama et al. described protective effects of EGCG against the trichothecene-induced cytotoxicity in mouse macrophages [47]. These results, despite needing further investigation, suggest that green tea components such as EGCG could be useful in protection against the toxic effects of mycotoxins eventually present in this beverage. Consequently, the low magnitude of risk identified in the present study could be even lower, if this referred potential protective effect of EGCG is confirmed.

According to our findings, the major limiting aspect influencing the risk of increasing the consumption of green tea is the potential adverse effects associated with high intake of EGCG (>800 mg EGCG/day). The current consumption of green tea in Portugal, ranging between 122.8 (less than one cup per day) and 458.9 g/day (around three cups per day), is not associated with an intake of EGCG being a health concern. Increasing the consumption of green tea to up to seven cups per day (corresponding to 1050 g/day) is expected to not cause health concern, and simultaneously, should maximize the beneficial effects associated with EGCG. Here, we considered just two green tea components—i.e., mycotoxins and EGCG. However, and in order to support future recommendations regarding green tea consumption in Portugal, a full quantitative risk–benefit assessment, considering additional components and the associated health effects, should be developed in the near future. However, and despite the uncertainties associated with the present assessment, mainly related with the low number of analyzed samples and EGCG levels not measured but assumed from the EFSA document [18], the present study opens the discussion to establish future research regarding the risk–benefit assessment of green tea, and consequently contributing to the increase in green tea consumption in Portugal.

## 4. Conclusions

In addition to the quantification of the occurrence levels of mycotoxins, the present study also assessed the balance between risks (due to mycotoxins and high levels of catechins) and benefits (due to moderate intake of catechins) associated with the consumption of green tea. Results evidenced that consumption of up to seven cups per day is expected to maximize the beneficial effects associated with green tea consumption, mainly associated with EGCG intake. Taking into consideration the potential beneficial effects, these results establish the basis for future research regarding the risk–benefit assessment of green tea as a tool contributing to support the definition of consumption recommendations of green tea. Future efforts should be dedicated to advancing the current evidence, through a full risk–benefit assessment, and subsequently support policy actions that aim to improve public health.

## 5. Materials and Methods

### 5.1. Sample Collection

Tea samples available to consumers were purchased from different markets in Lisbon. In order to ensure a representative sample of commercialized green tea, twenty different green tea samples were selected in different presentations: in bulk (10 samples) and in bags (10 samples). Considered samples were from seven different origins (China, Portugal (Azores), England, Japan, Indonesia, Ceylon (Sri Lanka), and Nepal).

### 5.2. Analytical Determination of Mycotoxins

Tea samples (1.0 g) were shaken with 4.0 mL of ACN:H2O:AcOH (79:20:1) for 60 min. After centrifugation for 5 min at 5000 rpm, raw extracts (0.5 mL) were diluted with 0.5 mL of water, mixed, centrifuged, and analyzed by the LC–MS/MS technique.

Mycotoxins were separated by a Nexera high performance liquid chromatograph (HPLC) (Shimadzu, Tokyo, Japan) using a Gemini C18 (150 × 4.6 mm, 5 μm) (Phenomenex, Torrance, CA, USA) chromatographic column with a flow rate of 1 mL/min and an injection volume of 5 μL. Mobile phases contained 5 mmol/L ammonium acetate and consisted of methanol/water/acetic acid 10/89/1 (*v*/*v*/*v*) (mobile phase A) and methanol/water/acetic acid 97/2/1 (*v*/*v*/*v*) (mobile phase B). The elution program was designed as follows: isocratic profile until 2.0 min with 0% of B, then from 2.0 to 5.0 min increase the organic phase to 50%, from 5.0 to 14.0 min increase the organic phase to 100%, from 14.0 to 18.0 min isocratic profile at 100% of B, and finally, from 18.0 min column equilibration for 4.5 min at 0% of B.

Mycotoxins were detected on a 5500 QTrap mass detector (Sciex, Foster City, CA, USA). Detection was performed in a single chromatographic run of both negative and positive polarities using scheduled Multiple Reaction Monitoring (sMRM) mode. The source parameters were as follows: curtain gas 30 psi, collision gas medium, ionspray voltage −4500 (negative polarity) and 5500 V (positive polarity), temperature 550 °C, ion source gas1 80 psi, and ion source gas2 80 psi. Mass spectrometry parameters of analyzed mycotoxins are presented in Table 2.

The detection (LOD) and quantification (LOQ) limits obtained for each mycotoxin are presented in Table 3.

### 5.3. Intake Assessment and Risk Estimates

Intakes of mycotoxins and catechins (particularly EGCG, the most relevant catechin in green tea) were estimated considering: (i) the obtained mycotoxins occurrence data (for the detected mycotoxins); (ii) the EGCG levels in green tea (as reported by the EFSA, 2018 [18]); (iii) European consumption data (for adults), both data according to the EFSA scientific opinion on the safety of green tea catechins [18]. Additionally, different hypothetical scenarios regarding the consumption of different number of cups (one cup of tea assumed as 150 mL), were considered to compute the risk of increasing the consumption of green tea, promoting the health benefits associated with catechins, avoiding risky values of exposure due to mycotoxin intake and/or excessive levels of catechins.

The instructions presented in the label of each product were considered to establish the amount of green tea present in each bag or the amount of green tea that is recommended to add to the water. In the absence of these values, the recommendations established by the ISO 3103 were followed [48].

To estimate the intake of mycotoxins, the highest levels quantified of the detected mycotoxins were considered. Mycophenolic acid was not included due to the inexistence of a reference value for this compound.

Regarding the EGCG, the intake values were compared with the no expected hepatotoxicity value (800 mg of EGCG per day), according to the EFSA (2018) [18]. EGCG levels were gathered by the EFSA and considered the determination of this compound in 100 samples of green tea [18]. It was assumed that intake levels below the referred no expected hepatotoxicity value were associated with health benefits.

For mycotoxins, margin of exposure (MOE) or hazard quotient (HQ) approaches were selected according to the genotoxic and/or carcinogenic potentials of the considered toxins. MOE was derived considering the ratio of lower confidence limit of the benchmark dose (BMDL10) and a MOE of 10,000 or more was considered to be of low concern for public health. For aflatoxin B1 and sterigmatocystin, the BMDL10 values considered were, respectively, 0.4 µg/kg bw/day [49] and 0.16 mg/kg bw/day [50]. For the HQ calculations, a ratio between the exposure levels and the tolerable daily intake (TDI) values were determined. A tolerable or a nontolerable exposure level was considered if HQ was below or above one, respectively. For fumonisin B1 and zearalenone, the TDI values considered were 0.1 (EFSA, 2018) and 0.25 µg/kg bw/day [51].

All the calculations were performed using the Microsoft^®^ Excel 2016.

## Figures and Tables

**Table 1:** Estimated intake and associated risk of mycotoxins and catechins (epigallocatechin-3-gallate (EGCG)). Highlighted values of risk (bold) represent intake above the levels considered as safe. A color code was used to express the concern for health: green color (risk corresponding to no health concern) and red color (risk corresponding to health concern).

Green Tea Consumption (g/Day)		Estimated Intake of Mycotoxins (ng/kg bw/Day) and Catechins (mg/Day)				Estimated Risk Associated with the Exposure to Mycotoxins and Catechins					
Current ^a^		AFB1	FB1	ZEA	STER	EGCG ^c^	AFB1 ^d^	FB1 ^e^	ZEA ^e^	STER ^d^	EGCG ^f^
Minimum	122.8	0.00002	0.0005	0.0003	0.00002	86.0	17,445,715	0.000005	0.000001	6,978,285,900	11
Maximum	458.9	0.00009	0.0019	0.0011	0.00009	321.2	4,669,857	0.000019	0.000004	1,867,942,679	40
**Hypothetical** ^b^											
1 cup/day	150	0.00003	0.0006	0.0004	0.00003	105	14,285,714	0.000006	0.000001	5,714,285,714	13
2 cups/day	300	0.00006	0.0013	0.0007	0.00006	210	7,142,857	0.000013	0.000003	2,857,142,857	26
3 cups/day	450	0.00008	0.0019	0.0011	0.00008	315	4,761,905	0.000019	0.000004	1,904,761,905	39
4 cups/day	600	0.00011	0.0025	0.0014	0.00011	420	3,571,429	0.000025	0.000006	1,428,571,429	53
5 cups/day	750	0.00014	0.0032	0.0018	0.00014	525	2,857,143	0.000032	0.000007	1,142,857,143	66
6 cups/day	900	0.00017	0.0038	0.0022	0.00017	630	2,380,952	0.000038	0.000009	952,380,952	79
7 cups/day	1050	0.00020	0.0044	0.0025	0.00020	735	2,040,816	0.000044	0.000010	816,326,531	92
8 cups/day	1200	0.00022	0.0050	0.0029	0.00022	840	1,785,714	0.000050	0.000011	714,285,714	**105**
9 cups/day	1350	0.00025	0.0057	0.0032	0.00025	945	1,587,302	0.000057	0.000013	634,920,635	**118**
10 cups/day	1500	0.00028	0.0063	0.0036	0.00028	1050	1,428,571	0.000063	0.000014	571,428,571	**131**

^a^ According to the EFSA, 2018 [18]. ^b^ Scenarios of consumption hypothetically assumed. 1 cup of green tea = 150 g. ^c^ EGCG levels assumed in accordance with the EFSA, 2018 [18]. ^d^ Margin of exposure (MOE) approach. MOE = BMDL10/Exposure data. ^e^ Hazard quotient (HQ) approach. HQ = intake values/reference values. ^f^ Percentage of intake compared to the EGCG level of no hepatotoxicity, according to the EFSA, 2018 [18]. AFB1 = aflatoxin B1; FB1 = fumonisin B1; ZEA = zearalenone; STER = sterigmatocystin; EGCG = epigallocatechin-3-gallate.

**Table 2:** Selected precursor and product ions of the analyzed mycotoxins with the respective declustering potential, collision energy, and cell exit potential values.

		Precursor Ion (m/z)	Product Ions (m/z)	Declustering Potential (V)	Collision Energy (V)	Cell Exit Potential (V)
15-Acetyldeoxynivalenol	[M+H]^+^	339.1	321.2/137.2	91	13/17	18/8
3-Acetyldeoxynivalenol	[M+Ac]^−^	397.3	59.2/307.1	−70	−38/−20	−8/−7
Aflatoxin B1	[M+H]^+^	313.1	285.2/128.1	106	33/91	16/10
Aflatoxin B2	[M+H]^+^	315.1	287.2/259.2	96	37/43	18/18
Aflatoxin G1	[M+H]^+^	329.1	243.1/200.0	86	39/59	14/12
Aflatoxin G2	[M+H]^+^	331.1	313.2/245.2	111	35/43	18/14
Aflatoxin M1	[M+H]^+^	329.1	273.2/229.1	91	35/59	16/12
α-Zearalanol	[M−H]^−^	321.2	277.2/303.2	−115	−32/−30	−13/−15
α-Zearalenol	[M−H]^−^	319.2	160.1/130.1	−115	−44/−50	−13/−20
β-Zearalanol	[M−H]^−^	321.2	277.2/303.2	−115	−32/−30	−13/−15
β-Zearalenol	[M−H]^−^	319.2	160.0/130.0	−115	−44/−50	−13/−20
Deepoxydeoxynivalenol	[M+Ac]^−^	339.1	59.1/249.0	−70	−20/−18	−9/−17
Deoxynivalenol	[M+Ac]^−^	355.1	265.2/59.2	−70	−22/−40	−13/−8
Diacetoxyscirpenol	[M+NH_4_]^+^	384.2	307.2/105.1	81	17/61	9/7
DON-3- Glucosid	[M+Ac]^−^	517.3	427.1/59.1	−80	−30/−85	−11/−7
Fumonisin B1	[M+H]^+^	722.5	334.4/352.3	121	57/55	4/12
Fumonisin B2	[M+H]^+^	706.5	336.4/318.4	126	59/51	8/2
Fumonisin B3	[M+H]^+^	706.5	336.3/318.5	126	59/51	8/2
Fusarenon-X	[M+Ac]^−^	413.2	59.1/263.0	−70	−44/−22	−9/−16
Gliotoxin	[M+H]^+^	327.1	263.2/245.3	61	15/25	16/20
Griseofulvin	[M+H]^+^	353.2	165.2/215.2	81	27/27	10/12
HT-2 Toxin	[M+NH_4_]^+^	442.2	263.1/345.1	76	21/27	19/20
Mevinolin	[M+H]^+^	405.3	199.2/173.3	76	17/29	14/10
Moniliformin	[M−H]^−^	96.9	41.2	−100	−24	−5
Monoacetoxyscirpenol	[M+NH_4_]^+^	342.2	265.1/307.2	71	13/13	26/8
Mycophenolic acid	[M+NH_4_]^+^	338.1	207.2/303.2	61	33/19	16/18
Neosolaniol	[M+NH_4_]^+^	400.2	215.0/185.0	76	25/29	12/14
Nivalenol	[M+Ac]^−^	371.1	281.1/59.1	−75	−22/−45	−15/−7
Ochratoxin A	[M+H]^+^	404.0	239.0/102.0	91	37/105	16/14
Ochratoxin B	[M+H]^+^	370.1	205.0/103.1	86	33/77	12/16
Patulin	[M−H]^−^	153.0	109.0/81.0	−50	−12/−18	−9/−11
Roquefortine C	[M+H]^+^	390.2	193.2/322.2	91	39/29	10/18
Sterigmatocystin	[M+H]^+^	325.1	310.2/281.1	96	35/51	18/16
T-2 Tetraol	[M+NH_4_]^+^	316.2	215.2/281.2	61	13/13	16/8
T-2 Toxin	[M+NH_4_]^+^	484.3	215.2/185.1	56	29/31	18/11
T-2 Triol	[M+NH_4_]^+^	400.2	281.3/215.2	71	13/17	16/12
Zearalanone	[M−H]^−^	319.2	205.2/107.0	−125	−34/−40	−13/−5
Zearalenon	[M−H]^−^	317.1	131.1/175.0	−110	−42/−34	−8/−13

**Table 3:** Mycotoxins measured and respective limits of detection (LODs) and quantification (LOQs) (ng/g).

Mycotoxins	LOQ	LOD
15-Acetyldeoxynivalenol	75.0	22.5
3-AcetylDON	22.9	6.9
Aflatoxin B1	2.4	0.7
Aflatoxin B2	1.5	0.5
Aflatoxin G1	1.7	0.5
Aflatoxin G2	3.1	0.9
Aflatoxin M1	2.8	0.9
Deepoxy-deoxynivalenol	63.1	19.0
Deoxynivalenol	66.5	20.0
Diacetoxyscirpenol	12.3	3.7
DON Glucoside	31.5	9.5
Fumonisin B1	42.4	12.7
Fumonisin B2	24.0	7.2
Fumonisin B3	29.9	9.0
Fusarenon-X	49.2	14.8
Gliotoxin	19.6	5.9
Griseofulvin	9.9	3.0
HT-2 Toxin	15.2	4.6
Mevinolin	8.0	2.4
Moniliformin	10.1	3.0
Monoacetoxyscirpenol	15.1	4.5
Mycophenolic acid	16.2	4.9
Neosolaniol	6.5	1.9
Nivalenol	35.1	10.5
Ochratoxin A	2.6	0.8
Ochratoxin B	4.2	1.3
Patulin	73.4	22.1
Roquefortine C	8.2	2.5
Sterigmatocystin	2.4	0.7
T2-Tetraol	32.8	9.8
T2-Toxin	7.1	2.1
T2-Triol	30.9	9.3
Zearalanone	5.5	1.7
Zearalenone	3.5	1.0
α-Zearalanol	7.7	2.3
α-Zearalenol	3.0	0.9
β-Zearalanol	12.5	3.7
β-Zearalenol	7.4	2.2

## Data Availability

Not applicable.
